# Discovery of Potential Anti-infective Therapy Targeting Glutamine Synthetase in *Staphylococcus xylosus*

**DOI:** 10.3389/fchem.2019.00381

**Published:** 2019-06-04

**Authors:** Wen-Qiang Cui, Qian-Wei Qu, Jin-Peng Wang, Jing-Wen Bai, God'spower Bello-Onaghise, Yu-Ang Li, Yong-Hui Zhou, Xing-Ru Chen, Xin Liu, Si-Di Zheng, Xiao-Xu Xing, Nsabimana Eliphaz, Yan-Hua Li

**Affiliations:** ^1^College of Veterinary Medicine, Northeast Agricultural University, Harbin, China; ^2^Heilongjiang Key Laboratory for Animal Disease Control and Pharmaceutical Development, Harbin, China; ^3^College of Science, Northeast Agricultural University, Harbin, China

**Keywords:** glutamine synthetase, molecular docking, sorafenib, anti-infective, mastitis

## Abstract

Glutamine synthetase (GS), which catalyzes the production of glutamine, plays essential roles in most biological growth and biofilm formation, suggesting that GS may be used as a promising target for antibacterial therapy. We asked whether a GS inhibitor could be found as an anti-infective agent of *Staphylococcus xylosus* (*S. xylosus*). Here, computational prediction followed by experimental testing was used to characterize GS. Sorafenib was finally determined through computational prediction. *In vitro* experiments showed that sorafenib has an inhibitory effect on the growth of *S. xylosus* by competitively occupying the active site of GS, and the minimum inhibitory concentration was 4 mg/L. *In vivo* experiments also proved that treatment with sorafenib significantly reduced the levels of TNF-α and IL-6 in breast tissue from mice mastitis, which was further confirmed by histopathology examination. These findings indicated that sorafenib could be utilized as an anti-infective agent for the treatment of infections caused by *S. xylosus*.

## Introduction

Mastitis is the most common and critical disease resulting in huge economic losses through the decreased production and milk quality in the global dairy cattle industry (Refaai et al., 2017; Chen et al., 2018). Furthermore, antimicrobial therapy for this disease has been increasingly challenging due to drug resistance (Virdis et al., 2010). Coagulase-negative staphylococci (CoNS) are mainly involved in the inflammatory processes of bovine udders (Pyörälä and Taponen, 2009; Marsilio et al., 2018). Notably, *Staphylococcus xylosus* (*S. xylosus*) accounts for about 40% of clinically isolated CoNS strains that cause cow mastitis (Klibi et al., 2018). Thus, novel antibacterial agents for the treatment of infections, including mastitis, caused by *S. xylosus* are urgently needed.

Glutamine synthetase (GS) has been described as an unusual multitasking protein that functions as an enzyme, a transcription coregulator, and a chaperone in ammonium assimilation as well as in the modulation and regulation of genes involved in biosynthesis of nitrogen and other important proteins (Schumacher et al., 2015). It is involved in the catalysis of ATP-dependent biosynthesis of glutamine from glutamate and ammonia (Murray et al., 2013). Nitrogen metabolism processes are linked to other metabolic networks *via* glutamine and glutamate (Liu et al., 2015). Glutamine production is essential for most biological growth and biomass production (Chandra et al., 2010). Remarkably, bacterial biofilm is generally controlled by metabolic processes, and nitrogen formation plays a key role in biofilm formation (Krajewski et al., 2008). Biofilms are a community of microorganisms that attaches to biological and non-biological surfaces. Biofilm-forming bacteria are 10–1,000 times more resistant to antimicrobial agents than planktonic cells (Mah and O'Toole, 2001) and have the ability to avoid phagocytosis by macrophages and neutrophils (Thurlow et al., 2011; Domenech et al., 2013), leading to recurrent infections or chronic inflammation. Therefore, GS inhibitors may represent a novel alternative in the control of bacterial infections.

**Figure 1:**
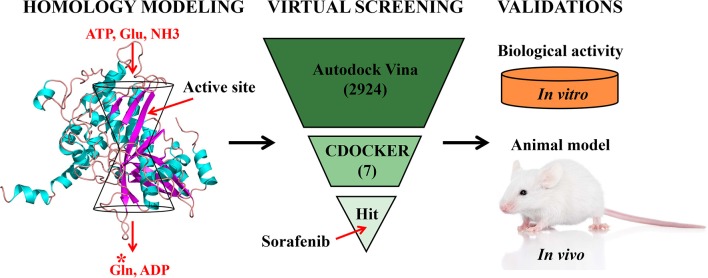
The screening work flow was applied to discover the novel GS inhibitor.

At present, studying cow mastitis by means of virtual screening of target protein GS has not been reported. We used computational prediction followed by experimental testing (Ung et al., 2016) to characterize GS. This current study is the first to demonstrate which unknown compound in the FDA database has antibacterial activity against *S. xylosus* infection *in vitro* and *in vivo*, opening a new direction for the treatment of dairy cow mastitis. The detailed screening work flow is shown in Figure 1.

## Materials and Methods

### Computational Prediction

#### Homology Modeling

The homology modeling technique is widely used to model proteins (Muhammed and Aki-Yalcin, 2019). Since the crystal structure of GS from *S. xylosus* is not available in the PDB database (https://www.rcsb.org/) at the moment, the construction of the 3D model of GS has become key to subsequent research. The amino acid sequence of GS was retrieved from the UniProt database (http://www.uniprot.org). In order to obtain an optimal template for the homology modeling, we did a sequence similarity search using NCBI-BLAST (http:/blast.ncbi.nlm.nih.gov/) to screen against the PDB database. Subsequently, the model was generated using EasyModeller 4.0 (Kuntal et al., 2010). The steps involved in this process were as previously described (Krieger et al., 2003): (i) The amino acid sequence of the selected template protein was identified by software. (ii) The sequence of GS was aligned to the template sequence(s). (iii) The backbone and loop modeling of the modeling were generated. (iv) The complete protein model of GS was formed and initially optimized.

#### Model Optimization and Evaluation

A protein model using homology modeling has been reported to produce unfavorable bond lengths, bond angles, torsion angles, and contacts. As a result, it was essential to minimize the energy to regularize local bond and angle geometry and to relax close contacts in the geometric chain (Messaoudi et al., 2013). Thus, the model was optimized and evaluated to verify the accuracy of the predicted GS structure. The model of GS was optimized using the Chiron server. Then, the model of GS was verified using the PROCHECK (Laskowski et al., 1993), ERRAT (Colovos and Yeates, 1993), and Verify 3D (Bowie et al., 1991) programs (http://servicesn.mbi.ucla.edu/SAVES/). The established structure was analyzed and visualized by UCSF Chimera. We aligned the structure of GS and its template to determine the active site of GS.

#### Virtual Screening

Structure-based virtual screening methods (Siddiquee et al., 2007) are important during the early stages of drug discovery, as they can screen compound databases using the active sites of proteins with known 3D structure (Gogoi et al., 2016). The ZINC15 database (Irwin and Shoichet, 2005) is an open source database and contains available chemical compound database (like the FDA database) prepared for virtual screening. The FDA database of listed compounds including a total of 2,924 compounds was downloaded. OpenBabel software converted the compounds in mol2 format to pdbqt format in batches using the script (Table S1a), ensuring that the virtual screening software AutoDock Vina (Trott and Olson, 2010) runs successfully. The processed compounds were used as the test by the batch processing script (Table S1b).

#### Molecular Docking

To avoid the drawbacks of rapid virtual screening and to obtain the starting structure of the GS/ligands complexes for molecular dynamics (MD) simulation, a standard docking procedure was carried out to find out whether these compounds have new binding modes with GS, so as to find new and more effective GS inhibitor lead compounds. Based on the results of virtual screening, docking studies were performed using CDOCKER (Wu et al., 2003), which adopts a flexible docking program based on CHARMM. During the docking calculations, all the protein residues were fixed and only the compound atoms were mobile. The module computes scores for each that best fits the potential target, which was termed as CDOCKER ENERGY. The best binding mode obtained was analyzed and visualized by PyMOL v2.0.6 software (Lill and Danielson, 2011).

#### Molecular Dynamics Simulation

The interactions between the GS/sorafenib complex were analyzed using MD simulation in Amber 16 (Case et al., 2005). The general AMBER force field (gaff) and ff14SB force field were used for the ligand and protein, respectively (Wang et al., 2005; Hornak et al., 2006). The energy minimization and equilibration protocol were done using the Sander program (Fu et al., 2018). The bond lengths involving hydrogen atoms were constrained using the SHAKE algorithm (Ryckaert et al., 1977). We determined the preferential binding mode of GS with sorafenib by 60-ns MD simulation based on the docking results. The equilibration of the trajectory was checked by monitoring the equilibration of quantities, such as the root-mean-square deviation (RMSD) with respect to the initial structure. The binding free energy Δ*G*_bind_ was calculated with the molecular mechanics and Poisson–Boltzmann solvation area (MM/PBSA) methodology for the GS/sorafenib complex based on stable MD trajectory. The free energy decomposition was performed to obtain the key residue contribution to the binding energy of sorafenib in the binding site of GS, analyzed using the MM-PBSA decomposition process. The binding interaction of the ligand–residue pair includes four terms: the Van der Waals contribution (Δ*E*_vdw_), the electrostatic contribution (Δ*E*_ele_), the solvation contribution (Δ*E*_sol_), and total contribution (Δ*E*_total_, Δ*E*_total =_ Δ*E*_vdw_ + Δ*E*_ele_ + Δ*E*_sol_). The energy components are calculated using the same snapshots as the free energy calculation. We choose a total number of 200 snapshots evenly from the last 10 ns on the MD trajectory. The specific details of the complex system have been previously described (Fu et al., 2018).

### *In vitro* Experiments

#### Bacterial Strains, Growth Conditions, and Reagents

*S. xylosus* ATCC 700404 was obtained from the American Model Culture Collection Library and grown at 37°C in TSB (Summus Ltd, Harbin, Heilongjiang, China); 98% pure sorafenib was purchased from Beijing Putian Tongchuang Biotech Company and was dissolved in 2.5% DMSO and used at the concentrations indicated.

#### Minimum Inhibitory Concentration

Minimum inhibitory concentration (MIC) was determined using broth microdilution (Garoff et al., 2018) in TSB medium. The overnight strains were grown in 5 ml TSB medium at 37°C. Then, the cultures of cells were diluted at a concentration of 1 × 10^5^ CFU/ml. Twenty microliters of sorafenib serially diluted in 2.5% DMSO was mixed with 180 μl of TSB containing bacteria in a 96-well microplate (Corning Costar® 3599 Corning, NY, USA). Sorafenib-free 2.5% DMSO mixed with 180 μl of TSB containing bacteria served as the control. The mixtures were incubated for 12 h at 37°C before visual reading. Test procedures and results were determined according to the standard microdilution method recommended by the Clinical and Laboratory Standards Institute (CLSI) (Fothergill, 2012).

#### Biofilm Formation

*S. xylosus* was evaluated for its capacity to form a biofilm. Two hundred microliters of cultures was distributed in a 96-well microplate containing 20 μl of sub-MICs of sorafenib solution. The drug concentrations were 1/2, 1/4, 1/8, and 1/16 MIC. The samples without sorafenib served as an untreated control. Similarly, sorafenib-free 2.5% DMSO was used to rule out the effect of solvent on biofilm formation. The specific details have been previously described (Zhou et al., 2018). Eventually, the values of OD at 590 nm were measured for each well using a microtiter plate reader (DG5033A, Huadong Ltd, Nanjing, China).

#### GS Inhibition Assay

*S. xylosus* was inoculated into TSB and sorafenib was added at a final concentration of 1/2 MIC (2 mg/L) in a 37°C incubator for 12 h. Control was incubated in the absence of sorafenib. GS test kit (Solarbio Biotechnology Co., Ltd, Beijing, China) was used to determine the activity of GS. The procedure was carried out according to the manufacturer's instructions. Briefly, the sample was centrifuged for 2 min at 12,000 rpm and the clear supernatant was discarded. Then, the bacterial pellet was washed twice with 1 ml of PBS and the supernatant was discarded. Two milliliters of the extract was added to a centrifuge tube containing the bacterial deposits. Bacteria were broken by an ultrasonic cell crusher (Xinzhi Biotechnology Co., Ltd, Ningbo, China). The samples were centrifuged for 10 min at 8,000 g/min at 4°C. The supernatant was collected and set on ice to be tested. Then, 200 μl of supernatant was taken to measure the absorbance at 540 nm in a 96-well microplate using a microtiter plate reader (DG5033A, Huadong Ltd, Nanjing, China).

#### Determination of Glutamine Content

The content of glutamine was measured using a glutamine test kit (A073, Institute of Biological Engineering, Nanjing, China). According to the manufacturer's instructions, the sample set on the ice from the above procedure was monitored at 630 nm using a UV-7504 ultraviolet spectrophotometer (Jingke Industrial Co., Ltd, Shanghai, China). Furthermore, protein concentrations were calculated by a BCA kit (Solaibao Biotechnology Co., Ltd, Beijing, China).

### *In vivo* Experiments

#### Animal

Twenty adult (10–12 weeks old) pathogen-free lactating BALB/c female mice that have given birth for 10 days with a mean weight of 30–32 g were purchased from the Experimental Animal Center of the Second Affiliated Hospital of Harbin Medical University. The mice were fed and given water *ad libitum*, and housed in a controlled environment. The light/dark cycle was 12 h/12 h, the light phase was from 06:00 to 18:00 GMT and the room temperature was 18–22°C. The mice were subjected to the experimental procedure after 3 days of rest. This study was approved and conducted in accordance with the guidelines of the Animal Welfare and Research Ethics Committee of Northeast Agricultural University. Welfare-related assessments and interventions were carried out prior to, during, or after the experiments (Kilkenny et al., 2010).

#### Treatment of Animals

The mice were randomly divided into four groups with five animals per group (normal group, model group, sorafenib group, and 2.5% DMSO group). The mice were separated from the offspring 12 h before being challenged. Those in the normal group were reared normally. In the model group, 100 μl of *S. xylosus* with a concentration of 10^9^ CFU/ml was injected into the fourth pair of mouse breasts. In the sorafenib group, the breasts were injected with 100 μl of sorafenib (MIC) after infection for 24 h. The 2.5% DMSO group served as the control.

#### Enzyme-Linked Immunosorbent Assay

Additional mammary gland tissues from the mice sacrificed at 24 h post-infection were homogenized in normal saline solution. The levels of the inflammatory cytokines TNF-α and IL-6 in the supernatants were measured using an enzyme-linked immunosorbent assay kit (Solaibao Biotechnology Co., Ltd, Beijing, China) according to the manufacturer's instructions. To further investigate the antibacterial activity of sorafenib, the samples were collected and processed as described above to determine the bacterial count by CFU/g.

#### Histopathological Examination

To evaluate the pathological correlates of mastitis, mammary gland tissues isolated from the sacrificed mice at 24 h post-infection were fixed in 4% paraformaldehyde and embedded in paraffin. Five-micrometer sections were prepared and stained with HE for histopathological examination and visualized by light microscopy.

#### Statistical Analysis

All the assays were performed in triplicate, and the results were expressed as means ± standard deviations. Statistics were determined using SPSS 19.0. Data were analyzed by the Student's *t*-test. The degree of statistically significance was reported as follows: ^*^p < 0.05, ^**^p < 0.01.

## Results and Discussions

### Computational Prediction

#### Homology Modeling and Verification

**Figure 2:**
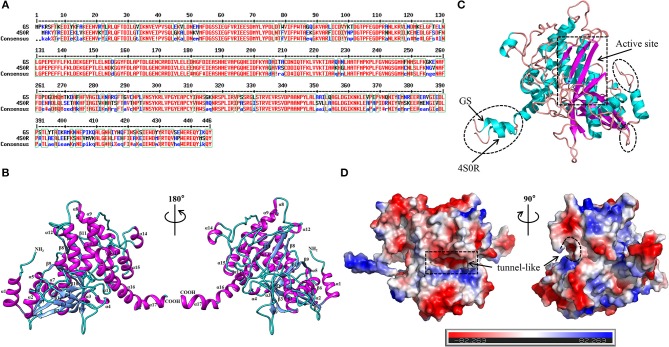
Determination of the 3D structure of the GS. **(A)** Sequence alignment between the target (GS) and template (PDB code: 4S0R). **(B)** The 3D structure of the GS. Cartoon diagrams of GS represented in the 180° rotation. The second structures were labeled (α-helices: magenta; β-strands: cornflower blue; random coils: cyan). The N terminus and the C terminus were also labeled. **(C)** Structural comparison of GS with 4S0R, highlighting their strong structural correspondence in the active site (rectangular dashed lines), and their differences were circled in elliptical dashed lines. **(D)** Electrostatic potential of the GS observed from different perspectives. The regions with negative or positive charge were colored red or blue, respectively. The catalytic site was marked by the dashed line.

The template protein used for homology modeling was the crystal structure (PDB code: 4S0R, with an identity similarity 75%) of *Bacillus subtilis (B. subtilis)* with a resolution of 3.5 Å. Generally, 30% sequence similarity to a known structure is considered to be the threshold limit for accurate homology modeling (Xiang, 2006), showing that it was the expected template of a highly similar sequence and reasonable for modeling and producing efficient structural models. For sequence alignment between GS (UniProt entry: A0A2T4PF96) and its template, see Figure 2A. The 3D structure of GS was carried out using UCSF Chimera (Figure 2B). As shown in Figure 2C, in making comparison between the structures of GS and its template while considering the binding site similarity, the quality of the structure in the binding site region, and the uniqueness of the conformation, the active region of the mimetic enzyme (*X*: 112.872, *Y*: 66.117, *Z*: −19.217, radius: 15 Å) was determined by superimposing proteins. Furthermore, a close comparison of GS and its target revealed that the RMSD differences were not significant (0.732 Å), suggesting that they have similar structure folds. The active site was a “tunnel-like” three-dimensional structure (Figure 2D), which limits the size of ligands and their binding modes.

The results of the PROCHECK, ERRAT, and Verify 3D programs are shown in Figures S1–S3. In summary, the verification results proved that the established 3D model of GS was a reasonable structure, which ensured that the subsequent virtual screening was accurate enough.

#### Virtual Screening

Based on the GS structure, a series of ligands from the FDA database were docked into the protein active site, and their binding affinities were estimated using AutoDock Vina. In Table 1, the optimal conformations with docking energy <-9.0 kcal/mol were selected in the docking search. The binding affinities between the GS and compounds were higher than those of all other compounds tested, implying that these compounds may have stronger inhibitory activity against GS. Hence, these candidate compounds were chosen for further interaction analysis in subsequent experiments.

**Table 1:** Lists of the top seven compounds obtained through virtual screening.

**Compound**	**ZINC number**	**Structure**	**Binding affinity**
1	ZINC14880002	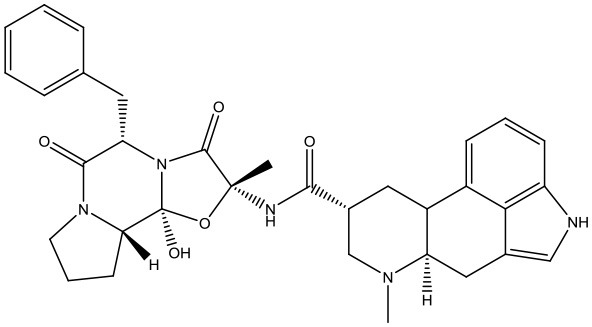	−9.7 kcal/mol
2	ZINC03978083	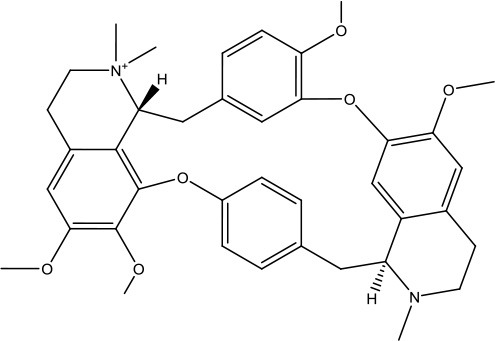	−9.5 kcal/mol
3	ZINC04097448	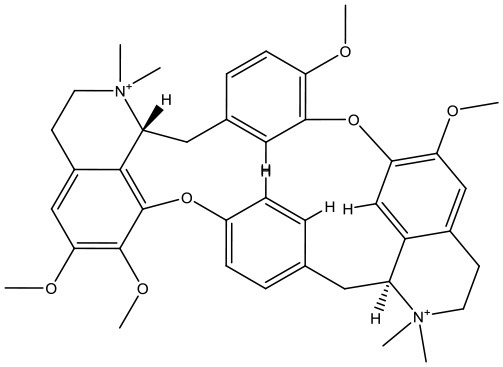	−9.4 kcal/mol
4	ZINC06716957	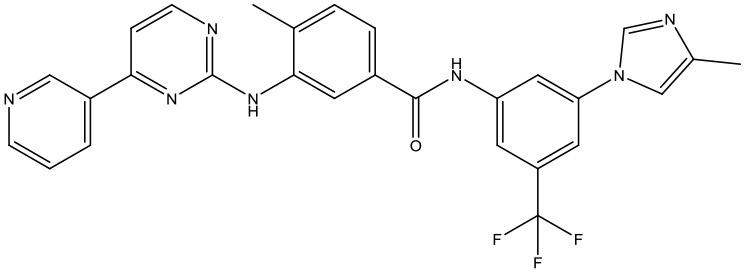	−9.2 kcal/mol
5	ZINC28240499	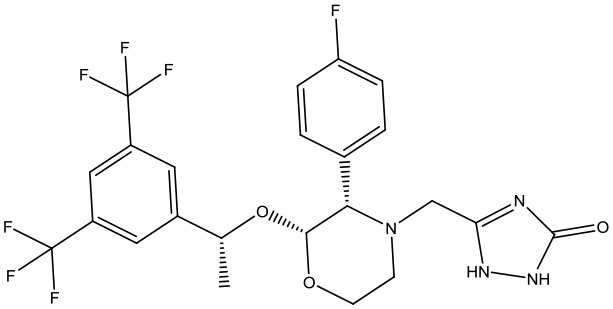	−9.1 kcal/mol
6	ZINC03927200	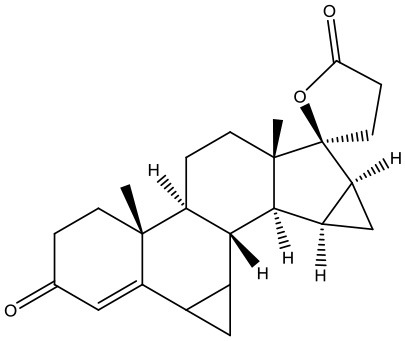	−9.0 kcal/mol
7	ZINC01493878	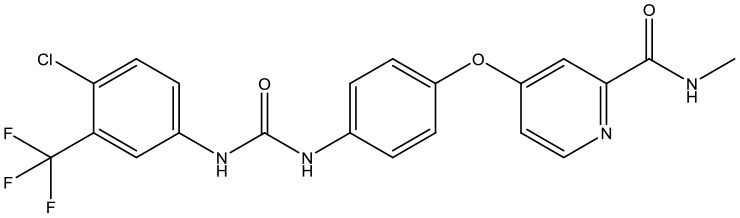	−9.0 kcal/mol

#### Molecular Docking

**Figure 3:**
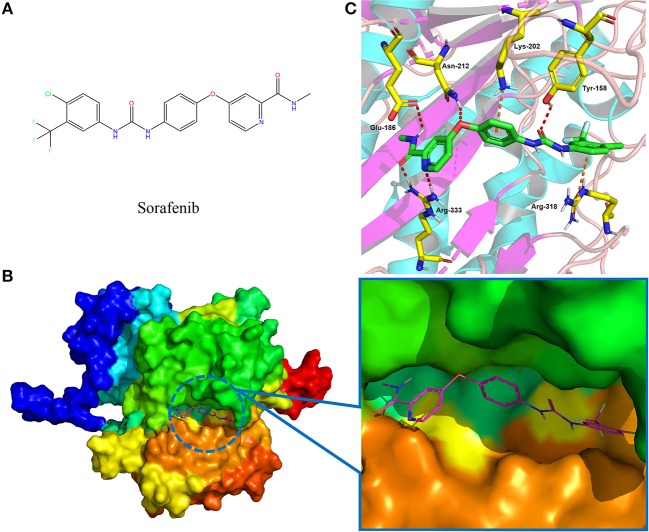
The complex structure model of sorafenib against the binding site of GS. **(A)** The molecular structure of the sorafenib. **(B)** Overall view of sorafenib at the tunnel-like active site. Sorafenib was shown in a magenta line model, and GS was shown in a rainbow surface model. **(C)** Detailed binding mode of sorafenib. Sorafenib was shown in a green stick model, and amino acid residues were shown in yellow sticks. Hydrogen bonds were shown in red dashed lines. Electrostatic interaction bonds were shown in orange dashed lines.

Following the docking simulations, it was observed that only sorafenib in the virtual screening hits (ZINC01493878, Figure 3A) bound with the active site and had the binding free energy (CDOCKER ENERGY: −13.7943 kcal/mol) of GS. The sizes of other small molecules except sorafenib were larger than that of sorafenib to fit into the active cavity, which may be one of the reasons for the unsuccessful docking in this follow-up docking. The potential building mode of the sorafenib with GS at the active cavity was explored in this study. Sorafenib was deeply embedded in the tunnel-like active area (Figure 3B). The molecule is relatively small, leaving room for further optimization toward higher potency (Shen et al., 2014). It was clear that sorafenib bound to GS *via* hydrogen bonds and electrostatic interactions with the amino acid residues Tyr-158, Glu-186, Asn-212, Arg-333, Arg-318, and Lys-202 (Figure 3C). Since the GS structural level of *S. xylosus* has not been studied yet, we first mentioned the key amino acids of the active site of the template protein (PDB code: 4S0R). Based on the known studies and analyses, amino acid residues Glu-184, Gly-241, Ser-249, Arg-298, Glu-304, Arg-316, and Arg-335 may have potential links with the catalytic substrates ATP and L-glutamate of the enzyme (Murray et al., 2013; Schumacher et al., 2015). As shown in Figure 1A, the key active amino acids Glu-186, Gly-243, Ser-251, Arg-300, Glu-306, Arg-318, and Arg-337 of the corresponding GS are aligned by sequence similarity. On the one hand, the directly interacting amino acids are Glu-186 and Arg-318, and they may be closely associated with ATP in the process of catalysis. On the other hand, these amino acids are very close in the entire active cavity. Hence, we conclude that the combination of sorafenib with other amino acids may also affect the catalysis of GS on its substrate.

#### Molecular Dynamics

**Figure 4:**
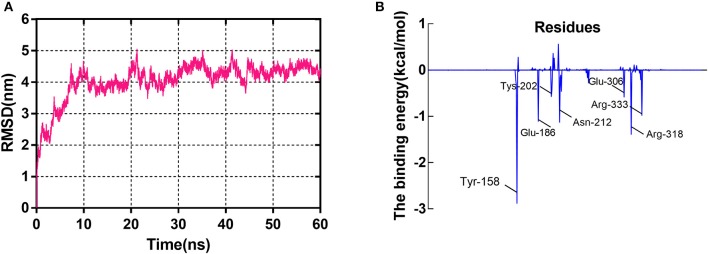
The molecular dynamics (MD) simulation of the GS/sorafenib complex. **(A)** The root-mean-square deviation (RMSD) calculated for the backbone atoms of the protein during MD simulation. **(B)** Decomposition of the binding energy on a per-residue basis in the binding sites.

Initially, the RMSD values of backbone C_α_ atoms are analyzed to examine whether each system reaches equilibrium, and it can be seen that the complex was found to reach equilibrium at 20 ns (Figure 4A). Figure 4A clearly shows that the RMSD values of the GS/sorafenib complex have a minor change from 20 to 60 ns. The overall results depict the stability of the protein in the simulation time because the RMSD values remain the same with little deviations. The MM/PBSA program was used to calculate the binding free energy from 50 to 60 ns. The Δ*E*_vdw_, Δ*E*_ele_, and Δ*G*_SA_ energy contributed favorably to Δ*G*_bind_, and the positive free energy (Δ*G*_PB/GB_) displayed adverse effect for the system. Δ*E*_ele_ made the greatest contribution to the binding free energy of the complex (Fu et al., 2018). Table 2 shows that Δ*G*_bind_ had a combined free energy value of −25.0237 kcal/mol. As shown in Figure 4B, Tyr-158, Glu-186, Asn-212, and Arg-318 had a strong total binding energy contribution, with Δ*E*_total_ of ≤-1.0 kcal/mol. The residues Tys-202, Glu-306, and Arg-333 also had an appreciable total binding energy contribution, with Δ*E*_total_ of ≤0.5–1.0 kcal/mol. These results suggest that these seven residues are key residues in the GS, except Glu-306, consistent with the CDOCKER docking results. Interestingly, as with the key amino acids mentioned in the molecular docking results, Glu-306 in the GS may be associated with the catalytic substrates L-glutamate of the enzyme. This fully demonstrated the reliability of molecular docking results. The docking displayed the binding orientation in the GS structure, increasing the confidence in our model and approach.

**Table 2:** Binding free energy of docked complex from the last 10 ns on the MD trajectory.

**Energy components**	**Energy**
Δ*E*_vdw_^a^	−32.1112 kcal/mol
Δ*E*_ele_^b^	−78.1970 kcal/mol
Δ*G*_PB/GB_^c^	90.6954 kcal/mol
Δ*G*_SA_^d^	−5.4109 kcal/mol
Δ*G*_bind_^e^	−25.0237 kcal/mol

a *Van der Waals energy*.

b *Electrostatic energy*.

c *Polar solvation energy with the PB model*.

d *Nonpolar solvation energy with the PB model*.

e *Free energy for binding (ΔG_bind_ = ΔE_vdw_ + ΔE_ele_ + ΔG_PB/GB_ + ΔG_SA_)*.

### *In vitro* Experiments

#### Anti-*S. xylosus* Activity of Sorafenib

**Figure 5:**
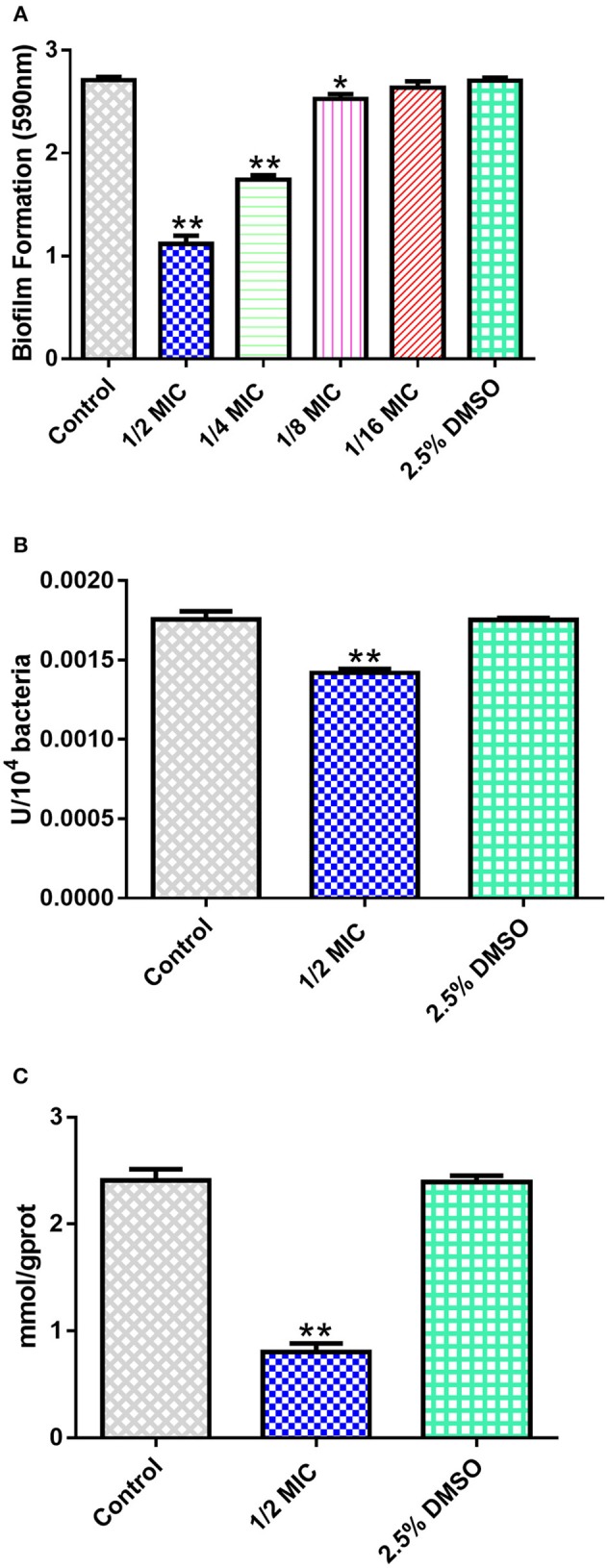
*In vitro* experiments. **(A)** Effect of sub-MICs of sorafenib on *S. xylosus* biofilm formation. **(B)** The inhibitory effect of 1/2 MIC sorafenib on GS activity. **(C)** The effect of 1/2 MIC sorafenib on the content of glutamine. The data were expressed as means ± SDs. ^*^p < 0.05, ^**^p < 0.01.

The MIC value for sorafenib against *S. xylosus* was 4 mg/L, indicating that sorafenib is a relatively highly efficient inhibitor of *S. xylosus*. In addition, we determined the effect of sub-MICs of sorafenib on biofilm production. As shown in Figure 5A, when compared with the control group, it was observed that the capacity of *S. xylosus* to form biofilm was significantly inhibited when the sorafenib concentration was 1/2 MIC (2 mg/L), 1/4 MIC (1 mg/L), and 1/8 MIC (0.5 mg/L) (*p* < 0.05). In contrast, there was no significant effect on the formation of *S. xylosus* biofilm at 1/16 MIC (0.25 mg/L) (*p* > 0.05). The effect of the solvent (2.5% DMSO) was also evaluated, and the results indicated that the 2.5% DMSO had no effect on biofilm formation (*p* > 0.05). These results revealed that sub-inhibitory concentrations of sorafenib could interfere with the biofilm formation of *S. xylosus*, suggesting that it may be a promising candidate drug in the inhibition of biofilm formation by *S. xylosus*. However, it is still unclear why sorafenib affected *S. xylosus* biofilm formation *in vitro*. Two pertinent questions were considered: (i) How does sorafenib affect *S. xylosus* biofilm formation at the enzyme level? (ii) Is it really related to the results of our computational prediction? Then, we followed up with a series of experiments below.

#### Sorafenib Inhibits GS Activity in *S. xylosus*

The change in enzyme activity was determined by OD value. As shown in Figure 5B, the activity of GS was significantly lower than that of the control group (*p* < 0.05). Furthermore, glutamine content was measured to indirectly reflect the activity of GS. It is noteworthy that a similar downward trend was observed when compared with the control group (Figure 5C). Considering the question raised earlier, is the intervention of *S. xylosus* on biofilm formation achieved by decreasing enzyme activity? In previous studies, GS was encoded by the *glnA* gene. The loss of the *glnA* gene results in the inability of *B. subtilis* to form biofilm on material surfaces, thereby weakening the pathogenicity of pathogens. It also affected cell surface characteristics by changing the chemical composition of the cell wall of *B. subtilis* (Liu et al., 2015). Interestingly, the structure of GS between *S. xylosus* and *B. subtilis* has a high degree of similarity (Figure 2C), so we have reasons to believe that, as we have predicted, sorafenib might be able to bind to the active site of GS, resulting in the reduction of GS activity and glutamine content. Thus, we hypothesized that treatment with sorafenib might inhibit the GS activity and subsequently triggers the reduction of biofilm formation in *S. xylosus*. It is worth mentioning that sorafenib is already an FDA-approved drug. It has been described as a multi-kinase inhibitor, and it is the first targeted drug that is known to improve the survival rate of patients with advanced hepatocellular carcinoma (Lang, 2008). Hence, the decrease of GS activity or the decrease of biofilm formation may have not only resulted from direct binding of sorafenib to GS. The results may also be related to the known multi-kinase inhibition (Lee et al., 2009).

### *In vivo* Experiments

#### Determination of TNF-α and IL-6 Inflammatory Factors

**Figure 6:**
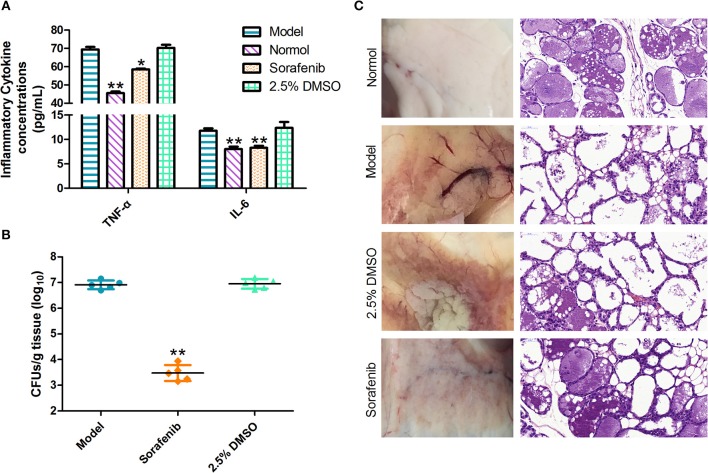
*In vivo* experiments. **(A)** Sorafenib reduces the inflammatory response in infected mice. **(B)** The effect of the sorafenib on the bacterial burden in infected mice. **(C)** Pathological changes in vision and histopathological analysis. The data were expressed as means ± SDs. ^*^p < 0.05, ^**^p < 0.01.

TNF-α is an important pro-inflammatory cytokine produced primarily by activated monocyte macrophages (Xu et al., 2006). IL-6 is a pro-inflammatory cytokine that stimulates B cells to produce a variety of inflammatory mediators involved in immune regulation. TNF-α and IL-6 are the most closely associated with inflammation (Winter and Colditz, 2002); they frequently reflect the incidence of mastitis by TNF-α and IL-6 detection methods (Guo et al., 2017). With regard to these observations (Figure 6A), the levels of cytokines, including TNF-α and IL-6, in the infected mice were markedly increased compared with those in the normal group, whereas administration of the sorafenib significantly reduced the *S. xylosus*-mediated increase in the inflammatory factors levels (*p* < 0.05). Consistent with these observations (Figure 6B), the bacterial counts in the mammary gland tissues indicated a significant decrease in the sorafenib group compared with the model group (*p* < 0.05). In addition, the 2.5% DMSO had no effect on the inflammatory cytokine concentration and bacterial counts (*p* > 0.05). These results showed that the immune cells were stimulated by the pathogenic microorganism to produce TNF-α. The immunoregulation of the sorafenib group inhibited the inflammatory reaction caused by the release of excess TNF-α, which was activated by mononuclear macrophages, and it had control effects on the concentration of IL-6 produced by activated macrophages. In previous studies, sorafenib has been approved by the FDA for the treatment of liver cancer (Lang, 2008). However, its clinical impact on the immune system is unclear. Other studies revealed that sorafenib also has immunomodulatory properties in addition to its anti-cancer effect at a minimal level of applications. It was reported that sorafenib-induced dendritic cells (SIDCs) can inhibit the proliferation of autologous T cells, thereby causing an alteration in their expression of cytokines including IL-1b, IL-2, IL-4, IL-10, IFN-g, IL-6, TNF-α, and CD25 (Zhao et al., 2016). This study was also in line with our findings on the inflammatory factors TNF-α and IL-6. Taken together, these results indicated that not only can sorafenib reduce inflammatory factors by inhibiting the growth of *S. xylosus*, but it may also act as an immunomodulator in the process.

#### Histopathological Examination

Following the challenge with *S. xylosus*, the breasts of lactating mice were injected with sorafenib to evaluate the impact of the treatment. Histopathological analysis of mammary gland tissues at 48 h post-infection was performed. As shown in Figure 6C, results revealed that the structure of the mammary gland was intact, and the epithelial cells were arranged neatly; no pathological change was observed in the normal group. In the model group, the acinar cavity of the mammary gland collapsed and was filled with blood coagulation, and there was a large amount of inflammatory cell infiltration. Besides, the intercellular substance was expanded and the epithelial detachment was seen. Interestingly, in the sorafenib group, the epithelium layer of the mammary gland was intact, and it was columnar or flat. The glandular cavity was highly dilated and filled with secretions. Moreover, apoptotic glandular epithelial cells were observed in the cavity, and inflammatory cell infiltration was intermittently seen in the stroma. There were no abnormalities in the 2.5% DMSO group. The results suggested that there was no pathological change in the mammary gland of mice in the model group before the mouse mastitis model was established. After 24 h of bacterial infection, inflammatory cells infiltrated the mammary gland in a large amount. After treatment with sorafenib, tissue repair, and regeneration were observed in injured tissues, thereby reducing the degree of breast lesions. From the results of our study, it can, therefore, be stated that treatment with sorafenib conferred effective protection against *S. xylosus*-induced mastitis in the mouse model. As we all know, sorafenib is connected with the known multi-kinase inhibition (Lee et al., 2009). In clinical applications, oral sorafenib was generally well-tolerated in patients with advanced hepatocellular carcinoma, with a manageable adverse event profile. However, it still shows certain side effects, such as diarrhea, hand, and foot skin, and so on (Keating, 2017). Actually, sorafenib shows cytotoxicity that could discourage its administration as an antibacterial agent, although there is a very positive effect on the mouse mastitis model. We consider that some pharmacology studies using a cow model will be used to evaluate efficacy and toxicity in order to explore the clinical application of sorafenib in the next in-depth study. Moreover, the treatment of cow mastitis is usually targeted by breast perfusion. On the one hand, some new pharmaceutical dosage forms can reduce the amount of drug used, like nano-formulations (Huang et al., 2018). We are going to develop sorafenib as a bulk drug into nano-formulations to improve drug targeting and then reduce the dose and the potential side effects of sorafenib. On the other hand, we hope to proceed with the modification of the structure of sorafenib itself, not only enhancing its affinity with GS but also reducing the production of toxicity.

## Conclusions

In conclusion, we successfully constructed a rational and hierarchical virtual screening flow to elucidate the potential inhibitor (sorafenib) for GS in *S. xylosus*. The *in vitro* study established that we evaluated the anti-*S. xylosus* activity of sorafenib. *In vivo*, treatment with the sorafenib remarkably alleviated *S. xylosus* pathological injury and inflammatory reactions in the mouse model of mastitis infection. Sorafenib has not been previously reported as a GS inhibitor. These results, therefore, provide an interesting template for designing new and more effective GS inhibitors in *S. xylosus*.

## Data Availability

The raw data supporting the conclusions of this manuscript will be made available by the authors, without undue reservation, to any qualified researcher.

## Ethics Statement

This study was approved and conducted in accordance with the guidelines of the Animal Welfare and Research Ethics Committee of Northeast Agricultural University. Welfare-related assessments and interventions that were carried out prior to, during, or after the experiment.

## Author Contributions

Y-HL designed the whole experiment. W-QC directed the completion of the experiment. Q-WQ, J-PW, J-WB, GB-O, Y-AL, Y-HZ, X-RC, XL, S-DZ, X-XX, and NE were supportive during the experiments.

### Conflict of Interest Statement

The authors declare that the research was conducted in the absence of any commercial or financial relationships that could be construed as a potential conflict of interest.
