# Supplementary material for: Discovery of Potential Anti-infective Therapy Targeting Glutamine Synthetase in Staphylococcus xylosus
Source: Front Chem. 2019 Jun 4;7:381. doi: 10.3389/fchem.2019.00381 (PMC6558069; doi:10.3389/fchem.2019.00381)
Supplement: Supplementary file 1 [file Data_Sheet_1.docx]

**Discovery of Potential Anti-infective Therapy Targeting Glutamine Synthetase in *Staphylococcus xylosus***

***Wen-Qiang Cui^1, 2^, Qian-Wei Qu^1, 2^, Jin-Peng Wang^1, 2^, Jing-Wen Bai^3^, Bello-Onaghise God’spower^1, 2^, Yu-Ang Li^1, 2^, Yong-Hui Zhou^1, 2^, Xing-Ru Chen^1, 2^, Xin Liu^1, 2^, Si-Di Zheng^1, 2^, Xiao-Xu Xing^1, 2^, Nsabimana Eliphaz^1, 2^, Yan-Hua Li^1, 2*^***

*^1^College of Veterinary Medicine, Northeast Agricultural University, Harbin, China,*

*^2^Heilongjiang Key Laboratory for Animal Disease Control and Pharmaceutical Development, Harbin, China,*

*^3^College of Science, Northeast Agricultural University, Harbin, China*

Correspondence author:

Professor Yanhua Li, College Veterinary Medicine, Northeast Agricultural University, 600 Changjiang Road, Xiangfang, Harbin, Heilongjiang 150030, P.R. China

Tel：+86 451 55191881

Email: liyanhua1970@163.com (Y-H.Li).

**Supplementary data**

**Table S1.** Scripts for virtual screening in Linux system.

| **Script** | **Procedure Code** |
| --- | --- |
| (a)^a^ | prepare_ligand.sh  for f in *.mol2;  do echo $f;  pythonadt ../script/prepare_ligand4.py -l $f -o $f.pdbqt -d ../etc/ligand_dict.py  Done |
| (b)^b^ | vina_screen_local.sh  for f in *.pdbqt; do  b=`basename $f .pdbqt`  echo Processing ligand $b  mkdir -p $b  vina --config conf.txt --ligand $f --out $/out.pdbqt --log $/log.txt  Done |

^a^ Convert the compounds mol2 format to pdbqt format in batches.

^b^ Obtain useful hits from ZINC15 database through structure-based approach.

**Figure S1.**

**
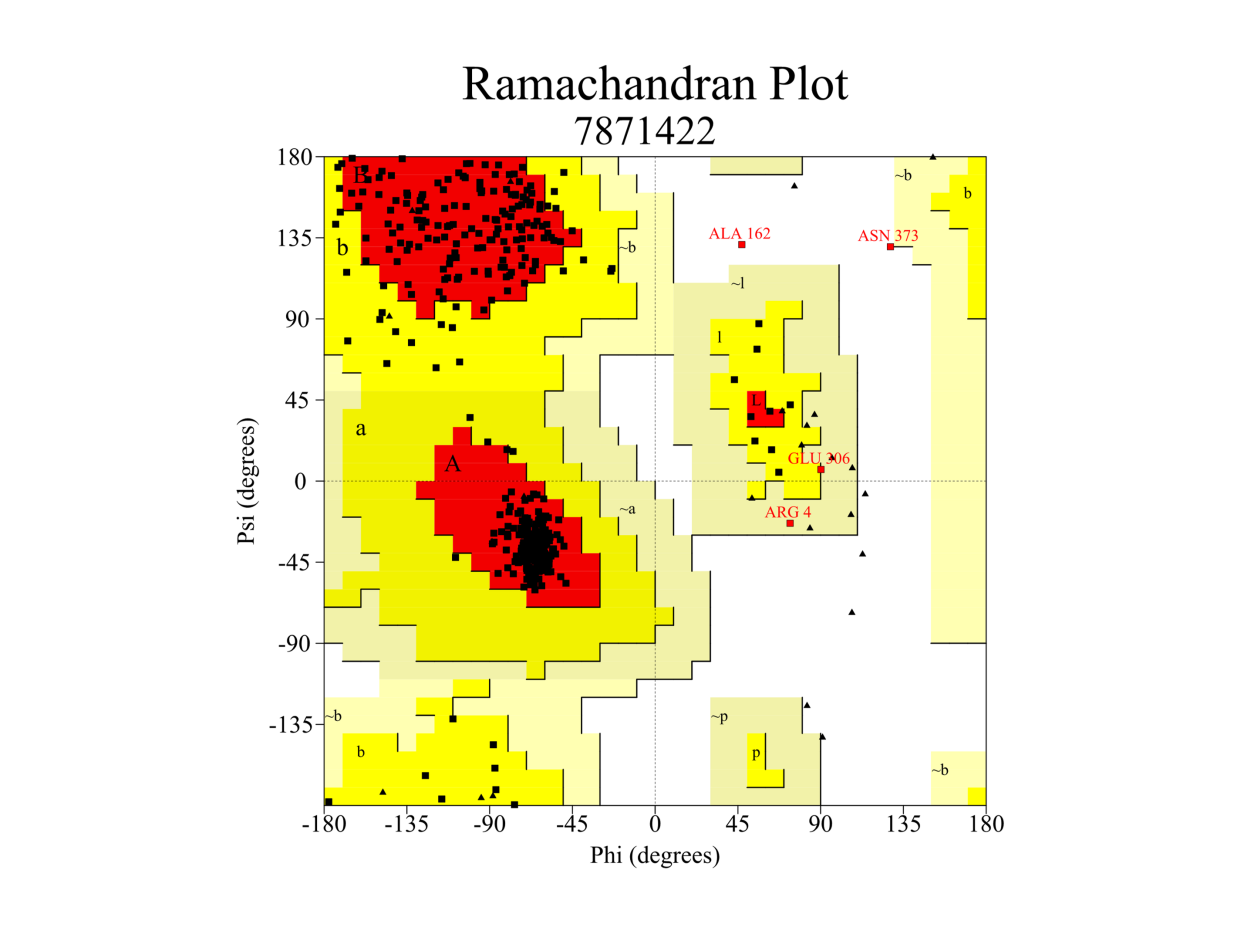
**

**Figure S1.** Ramachandran plot of GS obtained using PROCHECK software. Different colors indicate most favored regions (red), additional allowed regions (light yellow), generously allowed regions (dark yellow), disallowed regions (white). All of the 446 amino acid residues except 2 (0.5%) was in the disallowed region, which does not have a significant impact on our molecular simulations.

**Figure S2.**


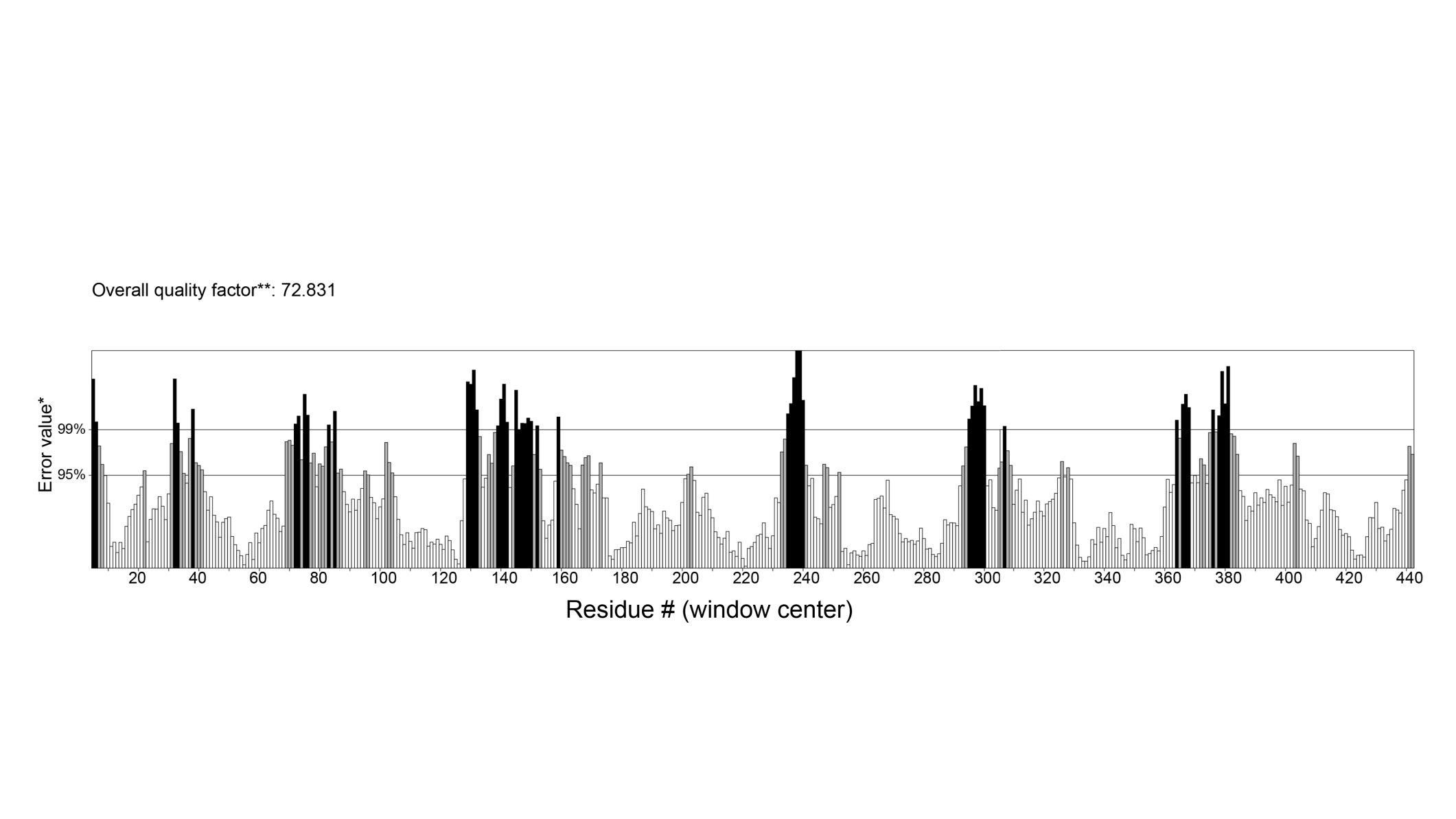


**Figure S2.** ERRAT Plot of GS. Two lines are drawn to indicate the confidence with which it is possible to reject regions that exceed that error value. **Expressed as the percentage of the protein for which the calculated error value falls below the 95% rejection limit. ERRAT is the overall quality factor of the non-bond atomic interaction. The higher the score, the higher the quality. The predicted overall quality factor was 72.831 for the GS 3D model. A generally accepted range is a high quality model greater than 50 ([Messaoudi et al., 2013](#_ENREF_2)).

**References**

Messaoudi, A., Belguith, H., and Ben Hamida, J. (2013). Homology modeling and virtual screening approaches to identify potent inhibitors of VEB-1 beta-lactamase. *Theoretical Biology and Medical Modelling* 10**,** 10. doi: 10.1186/1742-4682-10-22.

**Figure S3**


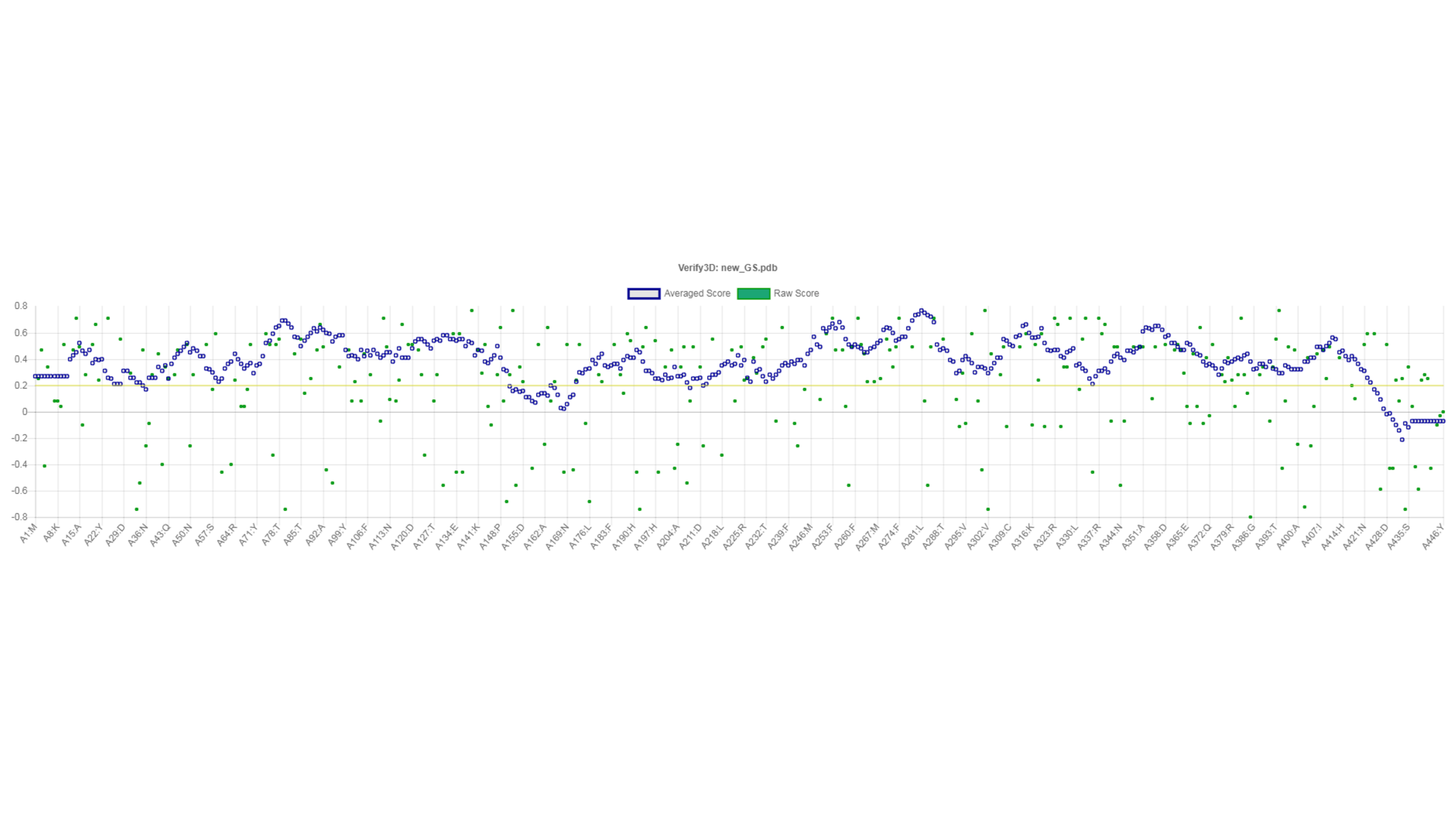


**Figure S3.** Calculated verify scores for residues of GS model by Verify-3D. Determines the compatibility of an atomic model (3D) with its own amino acid sequence (1D) by assigning a structural class based on its location and environment (alpha, beta, loop, non-polar etc) and comparing the results to good structures. For a better protein model, at least 80 % of the amino acids should have scored ≥ 0.2 in the 3D/1D profile. The Verify-3D results of GS showed that 89.91 % of the amino acids scored in the 3D/1D profile was 0.2.
